# GLIDE Global Health Ethics Leadership Awards 2025

**DOI:** 10.1177/15562646251403408

**Published:** 2025-12-08

**Authors:** Heidi Matisonn

**Affiliations:** 1 The EthicsLab, Department of Medicine and Neuroscience Institute, 37716 University of Cape Town, Cape Town, South Africa

**Keywords:** Research ethics, awards, glide, leadership, Oxford

Since its inception in 2022, the GLIDE Global Health Ethics Leadership Award, presented by the Global Infectious Disease Ethics Collaborative (a partnership between the Johns Hopkins Berman Institute for Bioethics and the Ethox Centre at the University of Oxford) has recognised individuals or teams for their ‘sustained contribution to the field of bioethics.’ Past recipients include Prof Nelson Sewankambo, Prof Debora Diniz and her research team, and Prof Bobbie Farsides. The 2025 recipients, Dr Barbara Sina, Dr Dan O’Connor and Ms Katherine Littler, were presented with their awards at the Oxford Global Health & Bioethics International Conference in July 2025 by conference chairs Prof Joseph Ali, Prof Jeffrey Kahn, Prof Patricia Kingori and Prof Michael Parker.

Dr Sina's work as Program Director of the Fogarty International Center in the National Institutes of Health where she has, for the last 25 years, catalysed the establishment and growth of over 60 research ethics training programs, across nearly 100 countries, has dramatically increased the capacity of individuals and institutions to address pressing research ethics challenges around the world – (see JERHRE special issues 8 Vol 5, Dec 2013 and 9 vol 2 Apr 2014 and forthcoming 2026 special issues on further Fogarty Program accomplishments). Dr O’Connor, who served as the Head of Research Environment until 30 June 2025, and prior to that from 2013 as the Head of Humanities and Social Science at Wellcome, was integral to the expansion of Wellcome's grant programs and he also created the first internal bioethics function at Wellcome, championing global health ethics research, education, and training. Currently the co-head of the Health Ethics & Governance Unit at the World Health Organization (WHO), Ms Katherine Littler led the Global Policy Team at Wellcome until 2018 and her leadership in both organizations and her re-establishment of the Global Forum for Bioethics in Research (GFBR, see Littler et al., 2014) has enabled bioethics scholars from both LMICs and HICs to meet collaboratively make a significant difference to research ethics policy and practice globally.

To underscore the extent of the contributions the three awardees have made to advancing research in bioethics and to the careers of bioethicists, audience members were asked to raise their hands if they had benefited from support from the Fogarty International Center (FIC) of the US National Institutes of Health, the Wellcome Trust, and/or the WHO. An overwhelming majority indicated that they had, and the standing ovations given to Dr Sina, Dr O’Connor, and Ms Littler served as further testament to just how much their contributions were appreciated.
